# (1S*,4′S*,5R*)-1-Isopropyl-5-meth­oxy-2′,3-dimethyl-4,6-dioxa-2-aza­spiro­[bicyclo­[3.2.0]hept-2-ene-7,4′-isoquinoline]-1′,3′(2′*H*,4′*H*)-dione

**DOI:** 10.1107/S1600536811015315

**Published:** 2011-04-29

**Authors:** Hoong-Kun Fun, Ching Kheng Quah, Chengmei Huang, Haitao Yu

**Affiliations:** a X-ray Crystallography Unit, School of Physics, Universiti Sains Malaysia, 11800 USM, Penang, Malaysia; b School of Chemistry and Chemical Engineering, Nanjing University, Nanjing, 210093, People’s Republic of China

## Abstract

In the isoquinoline ring system of the title mol­ecule, C_18_H_20_N_2_O_5_, the N-heterocyclic ring is in a half-boat conformation. The dioxa-2-aza­spiro ring is essentially planar, with a maximum deviation of 0.029 (1) Å, and makes a dihedral angle of 30.63 (5)° with the benzene ring. The mol­ecular structure is stabilized by a weak intra­molecular C—H⋯O hydrogen bond, which generates a *S*(6) ring motif. In the crystal, mol­ecules are linked *via* weak inter­molecular C—H⋯O hydrogen bonds into a three-dimensional supra­molecular network. Additional stabilization is provided by π–π stacking inter­actions between symmetry-related benzene rings with a centroid–centroid distance of 3.6507 (5) Å.

## Related literature

For general background to and the potential biological activity of the title compound, see: Pollers-Wieers *et al.* (1981 ▶); Malamas *et al.* (1994 ▶); Yu *et al.* (2010 ▶); Du *et al.* (2008 ▶); Chen *et al.* (2006 ▶); Zhang *et al.* (2006 ▶); Mitchell *et al.* (1995 ▶, 2000 ▶); Harris *et al.* (2005 ▶); Wang *et al.* (2010 ▶); Huang *et al.* (2011 ▶). For the stability of the temperature controller used in the data collection, see: Cosier & Glazer (1986 ▶). For standard bond-length data, see: Allen *et al.* (1987 ▶). For hydrogen-bond motifs, see: Bernstein *et al.* (1995 ▶). For ring conformations, see: Cremer & Pople (1975 ▶). 
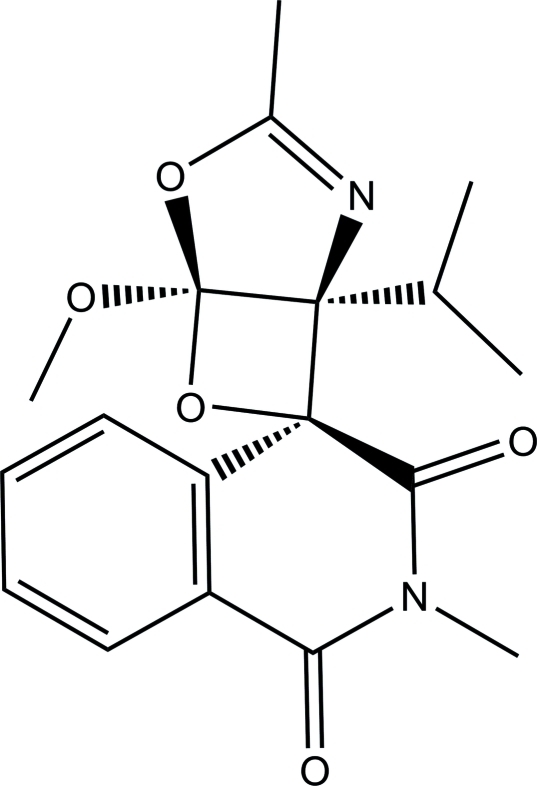


## Experimental

### 

#### Crystal data

C_18_H_20_N_2_O_5_
*M* *_r_* = 344.36
Monoclinic, 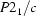

*a* = 10.5721 (2) Å
*b* = 10.4260 (2) Å
*c* = 15.7633 (3) Å
β = 101.641 (1)°
*V* = 1701.77 (6) Å^3^
*Z* = 4
Mo *K*α radiation
μ = 0.10 mm^−1^
*T* = 100 K
0.51 × 0.37 × 0.35 mm

#### Data collection

Bruker SMART APEXII CCD area-detector diffractometer
Absorption correction: multi-scan (*SADABS*; Bruker, 2009 ▶) *T*_min_ = 0.951, *T*_max_ = 0.966
23124 measured reflections
6242 independent reflections
5286 reflections with *I* > 2σ(*I*)
*R*_int_ = 0.026

#### Refinement

*R*[*F* ^2^ > 2σ(*F* ^2^)] = 0.040
*wR*(*F* ^2^) = 0.108
*S* = 1.02
6242 reflections
231 parameters
H-atom parameters constrained
Δρ_max_ = 0.49 e Å^−3^
Δρ_min_ = −0.21 e Å^−3^

### 

Data collection: *APEX2* (Bruker, 2009 ▶); cell refinement: *SAINT* (Bruker, 2009 ▶); data reduction: *SAINT*; program(s) used to solve structure: *SHELXTL* (Sheldrick, 2008 ▶); program(s) used to refine structure: *SHELXTL*; molecular graphics: *SHELXTL*; software used to prepare material for publication: *SHELXTL* and *PLATON* (Spek, 2009 ▶).

## Supplementary Material

Crystal structure: contains datablocks global, I. DOI: 10.1107/S1600536811015315/lh5237sup1.cif

Structure factors: contains datablocks I. DOI: 10.1107/S1600536811015315/lh5237Isup2.hkl

Additional supplementary materials:  crystallographic information; 3D view; checkCIF report

## Figures and Tables

**Table 1:** Hydrogen-bond geometry (Å, °)

*D*—H⋯*A*	*D*—H	H⋯*A*	*D*⋯*A*	*D*—H⋯*A*
C6—H6*A*⋯O2^i^	0.93	2.59	3.3754 (12)	143
C15—H15*A*⋯O5	0.96	2.56	3.2151 (12)	126
C18—H18*A*⋯O1^ii^	0.96	2.58	3.4298 (13)	148
C18—H18*C*⋯O2^iii^	0.96	2.58	3.3641 (12)	139

Symmetry codes: (i) 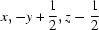; (ii) 
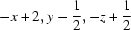
; (iii) 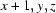.

**Fig. 1.**
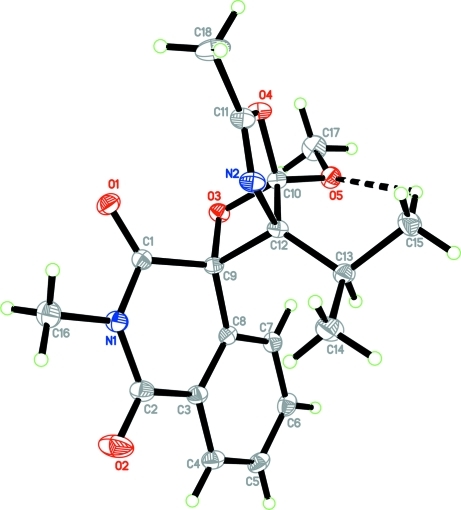
The molecular structure of the title compound showing 50% probability displacement ellipsoids for non-H atoms. Intramolecular interaction is shown as dash line.

**Fig. 2.**
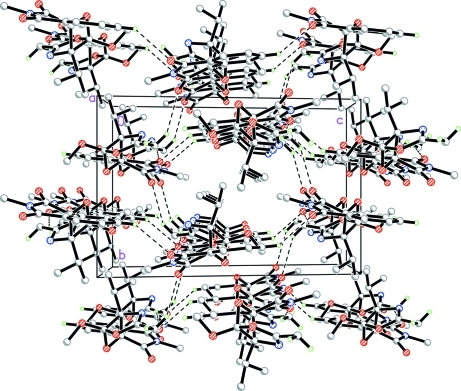
The crystal structure of the title compound, viewed along the a axis. H atoms not involved in hydrogen bonds (dashed lines) have been omitted for clarity.

## supplementary crystallographic information

### Comment

Isoquinolines are often found in bioactive natural products. They have been used to build blocks of benzo[c]phenanthridine alkaloids (Pollers-Wieers *et al.*, 1981; Malamas *et al.*, 1994; Yu *et al.*, 2010). Isoquinoline-1,3,4-trione derivatives were reported to be a type of small molecular inhibitor against caspase-3 which can promote apoptosis of the cells (Du *et al.*, 2008; Chen *et al.*, 2006). They can also attenuate apoptosis of neuronal cells induced by β-amyloid.(Zhang *et al.*, 2006). Isoquinoline-1,3,4-trione and its derivatives have been reported to be redox mediators of photosystems and have been used as herbicides (Mitchell *et al.*, 2000; 1995). Oxazole rings are also found in some bioactive natural products such as Annuloline and Ostreogrycin A. Oxazoles can be used to inhibit the activity of malignant tumors (Harris *et al.*, 2005). Since a lot of natural products especially the alkaloids containing the isoquinoline or oxazole ring are bioactive, there has been intense development of methodology to construct such moieties (Wang *et al.*, 2010). The title compound which was derived from isoquinoline-1,3,4-trione and oxazoles (Huang *et al.*, 2011) may have potential use in biochemical and pharmaceutical fields. Due to the importance of the isoquinoline-1,3,4-trione derivatives, we report herein the crystal structure of the title compound with a relative configuration of (1S^*^, 4'S^*^, 5R^*^).

In the title racemic compound, Fig. 1, atoms C9, C10 and C12 are the chiral centers. The isoquinoline ring system (N1/C1-C9) is not completely planar, the *N*-heterocyclic ring (N1/C1-C3/C8/C9) being distorted towards a half-boat conformation with atom C1 deviating by 0.243 (1) Å from the mean plane through the remaining atoms, puckering parameters (Cremer & Pople, 1975) Q = 0.2843 (9) Å, Θ =  64.10 (18)° and φ =100.97 (19)°. The dioxa-2-azaspiro ring (N2/O4/C10-C12) is essentially planar [maximum deviation of 0.029 (1) Å at atoms O4 and C10] and it inclines at a dihedral angle of 30.63 (5)° with the benzene ring (C3-C8). Bond lengths (Allen *et al.*, 1987) and angles are within normal ranges. The molecular structure is stabilized by a weak intramolecular C15–H15A···O5 hydrogen bond (Table 1) which generates a *S*(6) ring motif (Fig. 1, Bernstein *et al.*, 1995).

In the crystal structure, Fig. 2, molecules are linked *via* intermolecular C6–H6A···O2^i^, C18–H18A···O1^ii^ and C18–H18C···O2^iii^ hydrogen bonds (Table 1) into a three-dimensional supramolecular network. The crystal packing is further consolidated by π–π stacking interactions between the centroids of the C3-C8 (Cg1) rings, with a Cg1···Cg1^iv^ distance of 3.6507 (5) Å [symmetry code: (iv) 1-x, 1-y, -z].

### Experimental

The title compound was the main product from the photoreaction between isoquinoline-1,3,4-trione and 4-isopropyl-5-methoxy-2-methyloxazole. The compound was purified by flash column chromatography with ethyl acetate/petroleum ether (1:4) as eluents. X-ray quality crystals of the title compound was obtained from slow evaporation of an acetone and petroleum ether solution (1:5) (*m.p.* 451-453 K).

### Refinement

All H atoms were positioned geometrically and refined using a riding model with C–H = 0.93 or 0.98 Å and *U*_iso_(H) = 1.2 or 1.5 *U*_eq_(C). A rotating-group model was applied for the methyl groups.

### Crystal data

**Table:**

C_18_H_20_N_2_O_5_	*F*(000) = 728
*M**_r_* = 344.36	*D*_x_ = 1.344 Mg m^−^^3^
Monoclinic, *P*2_1_/*c*	Mo *K*α radiation, λ = 0.71073 Å
Hall symbol: -P 2ybc	Cell parameters from 9966 reflections
*a* = 10.5721 (2) Å	θ = 2.6–32.7°
*b* = 10.4260 (2) Å	µ = 0.10 mm^−^^1^
*c* = 15.7633 (3) Å	*T* = 100 K
β = 101.641 (1)°	Block, colourless
*V* = 1701.77 (6) Å^3^	0.51 × 0.37 × 0.35 mm
*Z* = 4	

### Data collection

**Table:**

Bruker SMART APEXII CCD area-detector diffractometer	6242 independent reflections
Radiation source: fine-focus sealed tube	5286 reflections with *I* > 2σ(*I*)
graphite	*R*_int_ = 0.026
φ and ω scans	θ_max_ = 32.7°, θ_min_ = 2.0°
Absorption correction: multi-scan (*SADABS*; Bruker, 2009)	*h* = −16→15
*T*_min_ = 0.951, *T*_max_ = 0.966	*k* = −10→15
23124 measured reflections	*l* = −23→23

### Refinement

**Table:**

Refinement on *F*^2^	Primary atom site location: structure-invariant direct methods
Least-squares matrix: full	Secondary atom site location: difference Fourier map
*R*[*F*^2^ > 2σ(*F*^2^)] = 0.040	Hydrogen site location: inferred from neighbouring sites
*wR*(*F*^2^) = 0.108	H-atom parameters constrained
*S* = 1.02	*w* = 1/[σ^2^(*F*_o_^2^) + (0.0531*P*)^2^ + 0.4865*P*] where *P* = (*F*_o_^2^ + 2*F*_c_^2^)/3
6242 reflections	(Δ/σ)_max_ = 0.001
231 parameters	Δρ_max_ = 0.49 e Å^−^^3^
0 restraints	Δρ_min_ = −0.21 e Å^−^^3^

### Special details

**Table:**

Experimental. The crystal was placed in the cold stream of an Oxford Cryosystems Cobra open-flow nitrogen cryostat (Cosier & Glazer, 1986) operating at 100.0 (1) K.
Geometry. All esds (except the esd in the dihedral angle between two l.s. planes) are estimated using the full covariance matrix. The cell esds are taken into account individually in the estimation of esds in distances, angles and torsion angles; correlations between esds in cell parameters are only used when they are defined by crystal symmetry. An approximate (isotropic) treatment of cell esds is used for estimating esds involving l.s. planes.
Refinement. Refinement of F^2^ against ALL reflections. The weighted R-factor wR and goodness of fit S are based on F^2^, conventional R-factors R are based on F, with F set to zero for negative F^2^. The threshold expression of F^2^ > 2sigma(F^2^) is used only for calculating R-factors(gt) etc. and is not relevant to the choice of reflections for refinement. R-factors based on F^2^ are statistically about twice as large as those based on F, and R- factors based on ALL data will be even larger.

### Fractional atomic coordinates and isotropic or equivalent isotropic displacement parameters (Å^2^)

**Table:**

	*x*	*y*	*z*	*U*_iso_*/*U*_eq_	
O1	0.83725 (6)	0.47548 (7)	0.19496 (4)	0.01927 (13)	
O2	0.43236 (7)	0.35091 (9)	0.21065 (5)	0.03015 (18)	
O3	0.80900 (6)	0.41336 (6)	0.02423 (4)	0.01457 (12)	
O4	1.01024 (6)	0.31366 (6)	0.07000 (4)	0.01661 (12)	
O5	0.88314 (6)	0.26083 (6)	−0.06194 (4)	0.01730 (13)	
N1	0.64083 (7)	0.39850 (8)	0.20730 (4)	0.01585 (14)	
N2	0.90028 (7)	0.20947 (7)	0.15999 (5)	0.01627 (14)	
C1	0.74047 (8)	0.41697 (8)	0.16262 (5)	0.01427 (14)	
C2	0.51566 (8)	0.36231 (9)	0.16792 (5)	0.01791 (16)	
C3	0.48885 (8)	0.34408 (8)	0.07267 (5)	0.01483 (15)	
C4	0.36037 (8)	0.32910 (9)	0.02963 (6)	0.01775 (16)	
H4A	0.2944	0.3288	0.0608	0.021*	
C5	0.33188 (9)	0.31473 (9)	−0.05953 (6)	0.01878 (16)	
H5A	0.2467	0.3038	−0.0883	0.023*	
C6	0.43059 (9)	0.31659 (9)	−0.10635 (5)	0.01847 (16)	
H6A	0.4110	0.3070	−0.1662	0.022*	
C7	0.55824 (8)	0.33279 (8)	−0.06381 (5)	0.01616 (15)	
H7A	0.6237	0.3353	−0.0953	0.019*	
C8	0.58806 (8)	0.34536 (8)	0.02622 (5)	0.01330 (14)	
C9	0.72502 (8)	0.35261 (8)	0.07458 (5)	0.01283 (14)	
C10	0.88181 (8)	0.29935 (8)	0.01999 (5)	0.01360 (14)	
C11	1.00499 (8)	0.26235 (9)	0.14963 (5)	0.01693 (15)	
C12	0.80770 (8)	0.22272 (8)	0.07841 (5)	0.01327 (14)	
C13	0.73890 (8)	0.09734 (8)	0.04853 (6)	0.01698 (15)	
H13A	0.6784	0.1133	−0.0063	0.020*	
C14	0.66170 (10)	0.04867 (9)	0.11444 (7)	0.02400 (19)	
H14A	0.5977	0.1111	0.1211	0.036*	
H14B	0.7190	0.0350	0.1692	0.036*	
H14C	0.6200	−0.0306	0.0944	0.036*	
C15	0.83697 (10)	−0.00346 (9)	0.03243 (7)	0.02421 (19)	
H15A	0.8840	0.0289	−0.0091	0.036*	
H15B	0.7923	−0.0805	0.0105	0.036*	
H15C	0.8960	−0.0218	0.0858	0.036*	
C16	0.66478 (10)	0.43923 (10)	0.29833 (5)	0.02245 (18)	
H16A	0.7481	0.4091	0.3275	0.034*	
H16B	0.5994	0.4041	0.3259	0.034*	
H16C	0.6625	0.5312	0.3012	0.034*	
C17	0.92704 (10)	0.35916 (10)	−0.11451 (6)	0.02420 (19)	
H17A	0.9248	0.3263	−0.1717	0.036*	
H17B	1.0138	0.3836	−0.0887	0.036*	
H17C	0.8715	0.4326	−0.1180	0.036*	
C18	1.12452 (9)	0.27703 (11)	0.21661 (6)	0.0258 (2)	
H18A	1.1267	0.2126	0.2604	0.039*	
H18B	1.1258	0.3605	0.2425	0.039*	
H18C	1.1984	0.2675	0.1904	0.039*	

### Atomic displacement parameters (Å^2^)

**Table:**

	*U*^11^	*U*^22^	*U*^33^	*U*^12^	*U*^13^	*U*^23^
O1	0.0165 (3)	0.0212 (3)	0.0188 (3)	−0.0030 (2)	0.0004 (2)	−0.0021 (2)
O2	0.0189 (3)	0.0524 (5)	0.0207 (3)	−0.0037 (3)	0.0078 (3)	0.0031 (3)
O3	0.0144 (3)	0.0141 (3)	0.0161 (2)	0.0000 (2)	0.0054 (2)	0.0029 (2)
O4	0.0117 (3)	0.0213 (3)	0.0163 (3)	−0.0018 (2)	0.0016 (2)	0.0039 (2)
O5	0.0197 (3)	0.0188 (3)	0.0144 (2)	−0.0020 (2)	0.0057 (2)	−0.0007 (2)
N1	0.0148 (3)	0.0204 (3)	0.0121 (3)	0.0003 (3)	0.0024 (2)	0.0008 (2)
N2	0.0133 (3)	0.0188 (3)	0.0162 (3)	0.0017 (3)	0.0017 (2)	0.0049 (2)
C1	0.0139 (3)	0.0147 (3)	0.0137 (3)	0.0012 (3)	0.0016 (3)	0.0012 (3)
C2	0.0150 (4)	0.0221 (4)	0.0165 (3)	0.0003 (3)	0.0028 (3)	0.0025 (3)
C3	0.0131 (3)	0.0149 (3)	0.0158 (3)	−0.0002 (3)	0.0013 (3)	0.0012 (3)
C4	0.0130 (4)	0.0179 (4)	0.0215 (4)	−0.0006 (3)	0.0015 (3)	0.0021 (3)
C5	0.0152 (4)	0.0159 (4)	0.0224 (4)	−0.0013 (3)	−0.0030 (3)	0.0006 (3)
C6	0.0200 (4)	0.0164 (4)	0.0165 (3)	0.0010 (3)	−0.0023 (3)	−0.0010 (3)
C7	0.0171 (4)	0.0162 (4)	0.0144 (3)	0.0016 (3)	0.0014 (3)	−0.0004 (3)
C8	0.0131 (3)	0.0117 (3)	0.0143 (3)	0.0003 (3)	0.0008 (3)	0.0010 (2)
C9	0.0119 (3)	0.0137 (3)	0.0128 (3)	−0.0008 (3)	0.0025 (2)	0.0015 (2)
C10	0.0120 (3)	0.0148 (3)	0.0138 (3)	−0.0005 (3)	0.0022 (2)	0.0009 (3)
C11	0.0150 (4)	0.0190 (4)	0.0164 (3)	0.0011 (3)	0.0020 (3)	0.0038 (3)
C12	0.0112 (3)	0.0140 (3)	0.0145 (3)	0.0002 (3)	0.0025 (2)	0.0023 (3)
C13	0.0147 (4)	0.0132 (3)	0.0233 (4)	−0.0006 (3)	0.0045 (3)	0.0011 (3)
C14	0.0217 (4)	0.0165 (4)	0.0366 (5)	−0.0006 (3)	0.0126 (4)	0.0057 (4)
C15	0.0219 (4)	0.0153 (4)	0.0372 (5)	0.0019 (3)	0.0104 (4)	−0.0001 (3)
C16	0.0236 (4)	0.0310 (5)	0.0125 (3)	0.0002 (4)	0.0033 (3)	−0.0012 (3)
C17	0.0298 (5)	0.0280 (5)	0.0174 (4)	−0.0049 (4)	0.0107 (3)	0.0023 (3)
C18	0.0161 (4)	0.0359 (5)	0.0224 (4)	−0.0038 (4)	−0.0032 (3)	0.0084 (4)

### Geometric parameters (Å, °)

**Table:**

O1—C1	1.2118 (10)	C7—H7A	0.9300
O2—C2	1.2176 (11)	C8—C9	1.4960 (11)
O3—C10	1.4250 (10)	C9—C12	1.6063 (12)
O3—C9	1.4504 (10)	C10—C12	1.5464 (11)
O4—C11	1.3759 (10)	C11—C18	1.4822 (12)
O4—C10	1.4336 (10)	C12—C13	1.5237 (12)
O5—C10	1.3553 (10)	C13—C14	1.5314 (13)
O5—C17	1.4520 (11)	C13—C15	1.5330 (13)
N1—C1	1.3937 (11)	C13—H13A	0.9800
N1—C2	1.3955 (11)	C14—H14A	0.9600
N1—C16	1.4686 (11)	C14—H14B	0.9600
N2—C11	1.2764 (11)	C14—H14C	0.9600
N2—C12	1.4569 (10)	C15—H15A	0.9600
C1—C9	1.5203 (11)	C15—H15B	0.9600
C2—C3	1.4830 (12)	C15—H15C	0.9600
C3—C8	1.3950 (11)	C16—H16A	0.9600
C3—C4	1.3991 (12)	C16—H16B	0.9600
C4—C5	1.3849 (12)	C16—H16C	0.9600
C4—H4A	0.9300	C17—H17A	0.9600
C5—C6	1.3947 (13)	C17—H17B	0.9600
C5—H5A	0.9300	C17—H17C	0.9600
C6—C7	1.3907 (12)	C18—H18A	0.9600
C6—H6A	0.9300	C18—H18B	0.9600
C7—C8	1.3965 (11)	C18—H18C	0.9600
			
C10—O3—C9	93.30 (6)	N2—C11—C18	126.18 (8)
C11—O4—C10	104.66 (6)	O4—C11—C18	115.10 (8)
C10—O5—C17	113.61 (7)	N2—C12—C13	112.66 (7)
C1—N1—C2	123.92 (7)	N2—C12—C10	104.11 (6)
C1—N1—C16	117.07 (7)	C13—C12—C10	121.79 (7)
C2—N1—C16	118.29 (7)	N2—C12—C9	112.13 (6)
C11—N2—C12	106.96 (7)	C13—C12—C9	119.31 (7)
O1—C1—N1	121.13 (7)	C10—C12—C9	83.07 (6)
O1—C1—C9	122.16 (7)	C12—C13—C14	111.17 (7)
N1—C1—C9	116.58 (7)	C12—C13—C15	110.04 (7)
O2—C2—N1	120.47 (8)	C14—C13—C15	110.86 (8)
O2—C2—C3	122.39 (8)	C12—C13—H13A	108.2
N1—C2—C3	117.09 (7)	C14—C13—H13A	108.2
C8—C3—C4	120.38 (7)	C15—C13—H13A	108.2
C8—C3—C2	121.32 (7)	C13—C14—H14A	109.5
C4—C3—C2	118.28 (8)	C13—C14—H14B	109.5
C5—C4—C3	119.69 (8)	H14A—C14—H14B	109.5
C5—C4—H4A	120.2	C13—C14—H14C	109.5
C3—C4—H4A	120.2	H14A—C14—H14C	109.5
C4—C5—C6	120.19 (8)	H14B—C14—H14C	109.5
C4—C5—H5A	119.9	C13—C15—H15A	109.5
C6—C5—H5A	119.9	C13—C15—H15B	109.5
C7—C6—C5	120.23 (8)	H15A—C15—H15B	109.5
C7—C6—H6A	119.9	C13—C15—H15C	109.5
C5—C6—H6A	119.9	H15A—C15—H15C	109.5
C6—C7—C8	119.97 (8)	H15B—C15—H15C	109.5
C6—C7—H7A	120.0	N1—C16—H16A	109.5
C8—C7—H7A	120.0	N1—C16—H16B	109.5
C3—C8—C7	119.53 (7)	H16A—C16—H16B	109.5
C3—C8—C9	119.08 (7)	N1—C16—H16C	109.5
C7—C8—C9	121.29 (7)	H16A—C16—H16C	109.5
O3—C9—C8	112.32 (6)	H16B—C16—H16C	109.5
O3—C9—C1	109.99 (6)	O5—C17—H17A	109.5
C8—C9—C1	113.71 (7)	O5—C17—H17B	109.5
O3—C9—C12	90.01 (6)	H17A—C17—H17B	109.5
C8—C9—C12	116.10 (6)	O5—C17—H17C	109.5
C1—C9—C12	112.35 (6)	H17A—C17—H17C	109.5
O5—C10—O3	113.67 (7)	H17B—C17—H17C	109.5
O5—C10—O4	111.35 (7)	C11—C18—H18A	109.5
O3—C10—O4	110.37 (6)	C11—C18—H18B	109.5
O5—C10—C12	121.22 (7)	H18A—C18—H18B	109.5
O3—C10—C12	93.42 (6)	C11—C18—H18C	109.5
O4—C10—C12	105.29 (6)	H18A—C18—H18C	109.5
N2—C11—O4	118.71 (7)	H18B—C18—H18C	109.5
			
C2—N1—C1—O1	162.97 (9)	C17—O5—C10—O4	−70.72 (9)
C16—N1—C1—O1	−7.11 (12)	C17—O5—C10—C12	164.67 (8)
C2—N1—C1—C9	−21.02 (12)	C9—O3—C10—O5	122.71 (7)
C16—N1—C1—C9	168.91 (7)	C9—O3—C10—O4	−111.37 (6)
C1—N1—C2—O2	−178.18 (9)	C9—O3—C10—C12	−3.67 (6)
C16—N1—C2—O2	−8.22 (14)	C11—O4—C10—O5	−138.23 (7)
C1—N1—C2—C3	−0.57 (13)	C11—O4—C10—O3	94.54 (7)
C16—N1—C2—C3	169.39 (8)	C11—O4—C10—C12	−5.10 (8)
O2—C2—C3—C8	−173.71 (9)	C12—N2—C11—O4	−1.58 (11)
N1—C2—C3—C8	8.73 (12)	C12—N2—C11—C18	177.19 (9)
O2—C2—C3—C4	8.11 (14)	C10—O4—C11—N2	4.53 (11)
N1—C2—C3—C4	−169.45 (8)	C10—O4—C11—C18	−174.37 (8)
C8—C3—C4—C5	0.38 (13)	C11—N2—C12—C13	132.03 (8)
C2—C3—C4—C5	178.58 (8)	C11—N2—C12—C10	−1.88 (9)
C3—C4—C5—C6	−0.71 (13)	C11—N2—C12—C9	−90.04 (8)
C4—C5—C6—C7	0.05 (13)	O5—C10—C12—N2	131.75 (8)
C5—C6—C7—C8	0.95 (13)	O3—C10—C12—N2	−107.82 (6)
C4—C3—C8—C7	0.61 (12)	O4—C10—C12—N2	4.38 (8)
C2—C3—C8—C7	−177.53 (8)	O5—C10—C12—C13	3.21 (12)
C4—C3—C8—C9	−175.90 (8)	O3—C10—C12—C13	123.64 (8)
C2—C3—C8—C9	5.95 (12)	O4—C10—C12—C13	−124.16 (8)
C6—C7—C8—C3	−1.27 (12)	O5—C10—C12—C9	−117.10 (8)
C6—C7—C8—C9	175.16 (8)	O3—C10—C12—C9	3.33 (5)
C10—O3—C9—C8	−114.88 (7)	O4—C10—C12—C9	115.52 (6)
C10—O3—C9—C1	117.40 (7)	O3—C9—C12—N2	99.19 (7)
C10—O3—C9—C12	3.52 (6)	C8—C9—C12—N2	−145.79 (7)
C3—C8—C9—O3	−152.15 (7)	C1—C9—C12—N2	−12.51 (9)
C7—C8—C9—O3	31.40 (10)	O3—C9—C12—C13	−125.97 (7)
C3—C8—C9—C1	−26.43 (10)	C8—C9—C12—C13	−10.95 (10)
C7—C8—C9—C1	157.12 (8)	C1—C9—C12—C13	122.33 (8)
C3—C8—C9—C12	106.23 (8)	O3—C9—C12—C10	−3.26 (5)
C7—C8—C9—C12	−70.21 (10)	C8—C9—C12—C10	111.76 (7)
O1—C1—C9—O3	−23.47 (11)	C1—C9—C12—C10	−114.96 (7)
N1—C1—C9—O3	160.56 (7)	N2—C12—C13—C14	61.23 (9)
O1—C1—C9—C8	−150.42 (8)	C10—C12—C13—C14	−174.06 (7)
N1—C1—C9—C8	33.61 (10)	C9—C12—C13—C14	−73.40 (9)
O1—C1—C9—C12	75.15 (10)	N2—C12—C13—C15	−61.98 (9)
N1—C1—C9—C12	−100.83 (8)	C10—C12—C13—C15	62.73 (10)
C17—O5—C10—O3	54.68 (9)	C9—C12—C13—C15	163.39 (7)

### Hydrogen-bond geometry (Å, °)

**Table:**

*D*—H···*A*	*D*—H	H···*A*	*D*···*A*	*D*—H···*A*
C6—H6A···O2^i^	0.93	2.59	3.3754 (12)	143
C15—H15A···O5	0.96	2.56	3.2151 (12)	126
C18—H18A···O1^ii^	0.96	2.58	3.4298 (13)	148
C18—H18C···O2^iii^	0.96	2.58	3.3641 (12)	139

Symmetry codes: (i) *x*, −*y*+1/2, *z*−1/2; (ii) −*x*+2, *y*−1/2, −*z*+1/2; (iii) *x*+1, *y*, *z*.
